# A simple and effective convolutional operator for node classification without features by graph convolutional networks

**DOI:** 10.1371/journal.pone.0301476

**Published:** 2024-04-30

**Authors:** Qingju Jiao, Han Zhang, Jingwen Wu, Nan Wang, Guoying Liu, Yongge Liu

**Affiliations:** 1 School of Computer and Information Engineering, Anyang Normal University, Anyang, Henan, China; 2 Key Laboratory of Oracle Bone Inscriptions Information Processing, Ministry of Education of China, Anyang, Henan, China; 3 School of Software Engineering, Anyang Normal University, Anyang, Henan, China; University of California Los Angeles, UNITED STATES

## Abstract

Graph neural networks (GNNs), with their ability to incorporate node features into graph learning, have achieved impressive performance in many graph analysis tasks. However, current GNNs including the popular graph convolutional network (GCN) cannot obtain competitive results on the graphs without node features. In this work, we first introduce path-driven neighborhoods, and then define an extensional adjacency matrix as a convolutional operator. Second, we propose an approach named *exop*GCN which integrates the simple and effective convolutional operator into GCN to classify the nodes in the graphs without features. Experiments on six real-world graphs without node features indicate that *exop*GCN achieves better performance than other GNNs on node classification. Furthermore, by adding the simple convolutional operator into 13 GNNs, the accuracy of these methods are improved remarkably, which means that our research can offer a general skill to improve accuracy of GNNs. More importantly, we study the relationship between node classification by GCN without node features and community detection. Extensive experiments including six real-world graphs and nine synthetic graphs demonstrate that the positive relationship between them can provide a new direction on exploring the theories of GCNs.

## 1. Introduction

Graph neural networks (GNNs) employ deep learning strategies to deal with graph-structured data and are applied to various fields [1], such as graph classification [2], recommender systems [3, 4] and natural language processing [5]. As a successful model of GNN, graph convolutional network (GCN) [6] has become a promising and important algorithm. In the past few years, many deuterogenic GCN and GNN algorithms have been proposed to resolve the problems, such as over fitting, over smoothing, high time complexity and poor performance. By randomly removing a certain number of edges in a graph at each training epoch, Rong et al propose an algorithm named Dropedge to resolve over fitting and over smoothing [7]. Eliasof et al propose a pathGCN model which learns a spatial operator from random paths on the graph to resolve the over smoothing problem [8]. In order to overcome the vanishing gradient problem generated by deep layers, Li et al bring residual/dense connections and dilated convolutions from convolutional neural networks (CNNs) into GCN architectures [9], and propose a deep GCN model that achieves 56 layers. Likewise, based on initial residual and identity mapping, Chen et al propose an extensional GCN called GCNII to build a deep GCN model [10]. Besides, GCNII also can relieve the problem of over smoothing. Because GCN suffers from the challenges of time and memory for training large graphs, Chen et al propose a FastGCN algorithm to resolve the problem mentioned above [11]. FastGCN first interprets graph convolutions as integral transforms, and then evaluates the integrals through Monte-Carlo approximation. Chiang et al propose a ClusterGCN algorithm to train very deep GCN on large-scale graphs [12]. The key strategy of ClusterGCN is that it samples a block of nodes from a dense subgraph in the input graph.

Furthermore, many algorithms have been proposed to improve the performance of GNNs. Fusing attention and multi-hop graph convolution model enables effective long-range message passing and improves the accuracies of GNNs [13, 14]. Wang et al propose a multi-hop attention graph neural network (MAGNA) to improve the performance of node classification [13]. MAGNA computes attention by aggregating the attention scores over all the possible multi-hop neighborhoods. Likewise, Xue et al present multi-hop hierarchical graph neural networks (MHGNNs) to obtain further node information and broad receptive field [14], and employ attention to extract significant hop-level features. By collecting the information from multi-hop neighboring nodes within one step of graph convolutions, Li et al propose a modified GNN to improve the accuracy of node classification [15].

In addition, some characters of GCN are researched. For example, Jin et al study the performance of GCN changes with different propagation mechanisms including 1-hop, 2-hop and *k*-nearest neighbor (*k*NN) network neighbors, and propose a U-GCN algorithm to improve the accuracy of GCN [16]. Jin et al have conducted research indicating GCN can destroy original node feature similarity which plays an important role in node classification. Therefore, they propose a framework named SimP-GCN to preserve node similarity while exploiting graph structure [17]. SimP-GCN can balance the information from graph structure and node features and achieve better performance on both assortative and disassortative graphs. Duong et al find that a strong correlation between node features and node labels may lead to better performance of GNN, and they propose new feature initialization methods to deal with non-attributed graphs [18]. Chen et al design a distribution matching named structure-attribute transformer (SAT) to deal with attribute-incomplete graphs. SAT, which achieves the joint distribution modeling of structures and attributes, can be used to link prediction and node attribute completion tasks [19]. Taguchi et al propose a varietal GCN to handle the graphs with incomplete (or missing) features that are dealt with Gaussian mixture model compensator [20]. The proposed method combines the processing of missing features with graph learning in a neural network architecture. Likewise, in the face of the graph with weak information (incomplete structure, incomplete features and insufficient labels), Liu et al design a dual-channel diffused propagation then transformation (D^2^PT) model to improve the performance of GNN [21]. D^2^PT enables GNN to propagate information for the nodes with long-scale range and the isolated nodes from the largest connected component.

Many graphs in the real world do not contain any feature information due to privacy concerns or difficulty in collecting node features [18]. An example is the social networks including REDDIT [22, 23] and Karate [24] data. This phenomenon also exists in the chemical field [25, 26]. However, the existing GNNs cannot achieve satisfactory performance on the graphs with incomplete features [19], and their performance deteriorates on the graphs without any node features [27]. In this work, we propose a simple and effective convolutional operator which enables GCN to achieve better performance on the graphs without node features. First, the proposed approach introduces an extensional adjacency matrix that is defined by the 2-path neighboring nodes as a convolutional operator. Then, the modified GCN named *exop*GCN is tested on six widely used graphs. Second, the proposed convolutional operator is applied to 13 GNN models, and the performances of most of these methods are improved significantly. At last, the relationship between node classification by GCN and community detection is studied. The experimental results show that *exop*GCN can offer superior performance over other GNNs in the graphs without node features, and also offer a general skill to improve accuracy of GNNs. More importantly, the results reveal that there is a strong correlation between node classification by GCN and community detection. It is expected that these results will open up a new venue for exploring the theories of GCNs.

## 2. Methods

### 2.1 Graph convolutional networks (GCNs)

Given an undirected and unweighted graph with *n* nodes and *m* edges, it can be described as G = (V,E), v = {*v*_*i*_|i = 1,2,⋯,*n*} is the set of nodes, and E = {*e*_*ij*_|i∈V and j ∈V} is the set of edges. The graph can also be described as an adjacency matrix **A**, if there is an edge between node *v*_*i*_ and node *v*_*j*_, then **A**_*ij*_ = 1; otherwise **A**_*ij*_ = 0. If each node *v*_*i*_ has *d* dimensional features, and all features of the nodes in the graph can be represented as a feature matrix **X** = [*x*_1_,*x*_2_,⋯,*x*_i_,⋯,*x*_n_]^*T*^∈*R*^*n*×*d*^.

Graph convolutional network (GCN) [6] is a typical and successful model of GNN and is applied to many fields. The main reason for the success of GCN is its ability to effectively aggregate the feature information of neighboring nodes by adjacency matrix **A** (see Eq (1)). To balance the features of neighboring nodes and self-node and to prevent the values of the nodes with high degree being too large in multi-convolutional layers, GCN uses modified convolutional matrix to aggregate the feature information (see Eq (2)).

Y=AX (1)

$$\tilde{Y}={\tilde{D}}^{-1/2}\tilde{A}{\tilde{D}}^{-1/2}X$$ (2)

where **Y** and $\tilde{Y}$ are feature matrixes, $\tilde{A}=A+I$ and **I** is an identity matrix, $\tilde{D}$ is a degree matrix, ${\tilde{D}}_{ii}=\sum_{j=1}^{N}{\tilde{A}}_{ij}$.

Using the convolutional matrix $\hat{A}={\tilde{D}}^{-1/2}\tilde{A}{\tilde{D}}^{-1/2}$, the layer-wise propagation rule for GCN is described as Eq (3).

$$H^{\left(l+1\right)}=\sigma ({\tilde{D}}^{-1/2}\tilde{A}{\tilde{D}}^{-1/2}H^{\left(l\right)}W^{\left(l\right)})$$ (3)

where **H**^(*l*)^ is the matrix of activations in the *l*^*th*^ layer; **H**^(0)^ = **X**,σ(⋯) denotes an activation function, such as the *ReLU*(⋯) = *max*(0,⋯); **W**^(*l*)^∈*R*^*d*×*f*^ with *d* dimensional feature vector and *f* filters is a trainable weight matrix in the layer *l*.

GCN considers a two-layer for semi-supervised node classification on a graph based on the layer-wise propagation rule. The forward model of GCN is represented by Eq (4).

$$Z=softmax(\hat{A}ReLU(\hat{A}XW^{\left(0\right)})W^{\left(1\right)})$$ (4)

where $\hat{A}={\tilde{D}}^{-1/2}\tilde{A}{\tilde{D}}^{-1/2},softmax(x_{i})=\frac{1}{\sum_{i}exp(x_{i})}exp(x_{i})$, the weights **W**^(0)^ and **W**^(1)^ are trained using gradient descent. The loss function is defined as the cross-entropy error over all labeled nodes (Eq (5)):

$$L=-\sum_{l\in y_{L}}\sum_{f=1}^{F}Y_{lf}\ln Z_{lf}$$ (5)

where *y*_*L*_ is the set of node indices with labels, *F* is the dimension of the output features and is equal to the number of classes. *Y*∈*R*^|*y*_*L*_|×*F*^ is a label indicator matrix.

### 2.2 The proposed method *exop*GCN

**Fig 1:**
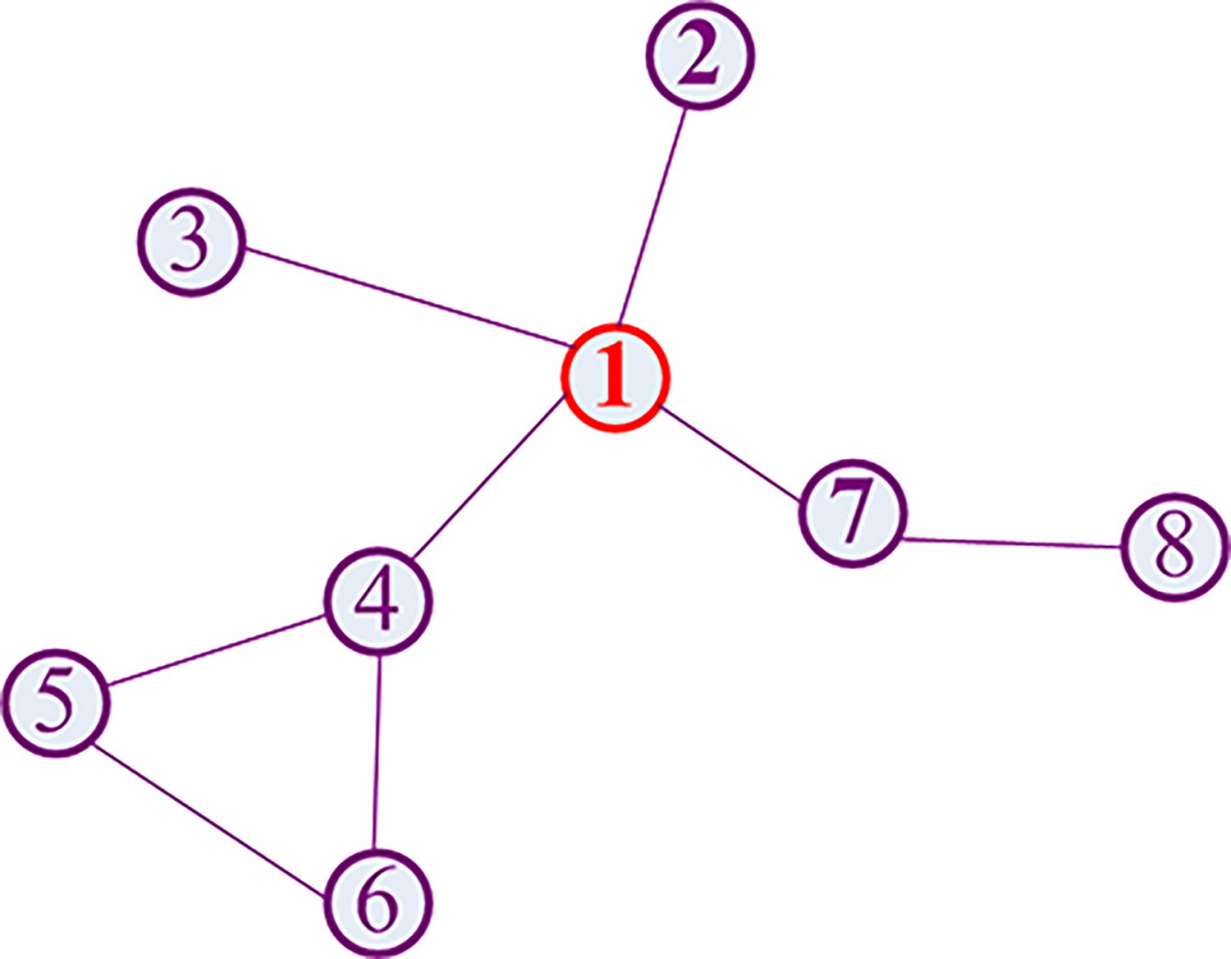
An example of graph for GCN.

In this section, we analyze the process of aggregation of neighboring nodes by GCN in detail. We can rewrite Eq (1) in the form of matrix (Eq (6)). In Eq (6), **Y** represents the feature matrix that is generated by graph convolution, and $Y_{ig}^{k}$ represents the *g*th feature of node *v*_*i*_ in the convolutional layer *k*. We take a small graph (see Fig 1) for example, assuming the node *v*_1_ has four neighboring nodes (*v*_2_,*v*_3_,*v*_4_,*v*_7_), that is *N*(*v*_1_) = {*v*_2_,*v*_3_,*v*_4_,*v*_7_}, and *N*(*v*_2_) = {*v*_1_}, *N*(*v*_3_) = {*v*_1_}, *N*(*v*_4_) = {*v*_1_,*v*_5_,*v*_6_}, *N*(*v*_7_) = {*v*_1_,*v*_8_}, respectively.


$$\left[\begin{array}{ccc} Y_{11} & \cdots & Y_{1d}\\ \vdots & \ddots & \vdots \\ Y_{n1} & \cdots & Y_{nd} \end{array}\right]=\left[\begin{array}{ccc} A_{11} & \cdots & A_{1n}\\ \vdots & \ddots & \vdots \\ A_{n1} & \cdots & A_{nn} \end{array}\right]\times \left[\begin{array}{ccc} X_{11} & \cdots & X_{1d}\\ \vdots & \ddots & \vdots \\ X_{n1} & \cdots & X_{nd} \end{array}\right]$$ (6)

In the first convolutional layer, the first feature ($Y_{11}^{1}$) of node *v*_1_ is aggregated by *N*(*v*_1_) in Eq (7). Likewise, the values of $Y_{21}^{1},Y_{31}^{1},Y_{41}^{1}$ and $Y_{71}^{1}$ are calculated by Eqs (8)–(11) respectively. From Eqs (7)–(11), it can be observed that GCN only captures the features of neighboring nodes (1-path neighboring nodes, see Eq (16)). Following, we calculate the features in the second convolutional layer of GCN by Eq (12). The feature $Y_{11}^{2}$ in the second layer can capture 2-path neighboring features (nodes *v*_5_,*v*_6_ and *v*_8_).


$$Y_{11}^{1}=X_{21}+X_{31}+X_{41}+X_{71}$$ (7)

$$Y_{21}^{1}=X_{11}$$ (8)

$$Y_{31}^{1}=X_{11}$$ (9)

$$Y_{41}^{1}=X_{11}+X_{51}+X_{61}$$ (10)

$$Y_{71}^{1}=X_{11}+X_{81}$$ (11)

$$Y_{11}^{2}={4X}_{11}+X_{51}+X_{61}+X_{81}$$ (12)

Here, we mainly consider the nodes without feature. For simplicity, we do not employ Eq (2) to deal with adjacency matrix **A**. If the nodes in the graph do not have any feature, GCN employs identity matrix (**I**) to replace the feature matrix (Eq (13)). For example, we can see that the value of ${\left(Y_{11}^{1}\right)}^{without}$ is 0 in the first convolutional layer (see Eq (14)), and (**Y**^1^)^*without*^ = **A**. In the second convolutional layer, the value of ${\left(Y_{11}^{2}\right)}^{without}$ is calculated by 1-path neighboring nodes (see Eq (15)). Note that, the calculation of ${\left(Y_{11}^{2}\right)}^{without}$ is determined by the value of 1-path neighboring nodes because some elements in (**Y**^1^)^*without*^ are equal to 0. Like the case with node features, ${\left(Y_{11}^{2}\right)}^{without}$ also captures the information of 2-path neighboring nodes. Because long-range propagation can effectively improve the performance of GNNs [21]. Therefore, is there a method for GCN to propagate deeper long neighboring nodes with two convolutional layers.


$$\left[\begin{array}{ccc} {(Y_{11)}}^{without} & \cdots & {\left(Y_{1n}\right)}^{without}\\ \vdots & \ddots & \vdots \\ {\left(Y_{n1}\right)}^{without} & \cdots & {\left(Y_{nn}\right)}^{without} \end{array}\right]=\left[\begin{array}{ccc} A_{11} & \cdots & A_{1n}\\ \vdots & \ddots & \vdots \\ A_{n1} & \cdots & A_{nn} \end{array}\right]\times \left[\begin{array}{ccc} 1 & \cdots & 0\\ \vdots & \ddots & \vdots \\ 0 & \cdots & 1 \end{array}\right]$$ (13)

$${\left(Y_{11}^{1}\right)}^{without}=0$$ (14)

$${\left(Y_{11}^{2}\right)}^{without}=A_{12}\times A_{12}+A_{13}\times A_{13}+A_{14}\times A_{14}+A_{17}\times A_{17}$$ (15)

To solve this problem, this work proposes a modified GCN named *exop*GCN for node classification without features. *exop*GCN first introduces an extensional adjacency matrix by path-driven neighboring nodes [27], and then a convolutional operator is performed on GCN for node classification without features. The path-driven neighboring nodes (or called *t*-path neighboring nodes) $N_{i}^{t}$ of node *v*_*i*_ is defined by the shortest path between two nodes, that is $N_{i}^{t}$ is the sets of nodes whose shortest path (*d*_*sp*_) to node *v*_*i*_ is less than or equal to *t* (see Eq (16)). Based on the definition of path-driven neighboring nodes, we can construct the extensional adjacency matrix **M**^*t*^ and the element in $M_{ij}^{t}$ is defined by Eq (17).


$$N_{i}^{t}=\left\{ \begin{array}{ll} {\{v_{j}|v}_{j}\in V,\;i\neq j\;and\;d_{sp}(i,j)\leq t\} & t>0\\ NULL & t=0 \end{array}\right.$$ (16)

$$M_{ij}^{t}=\left\{ \begin{array}{rr} 1 & i\neq jandj\in N_{i}^{t}\\ 0 & otherwise \end{array}\right.$$ (17)

**Fig 2:**
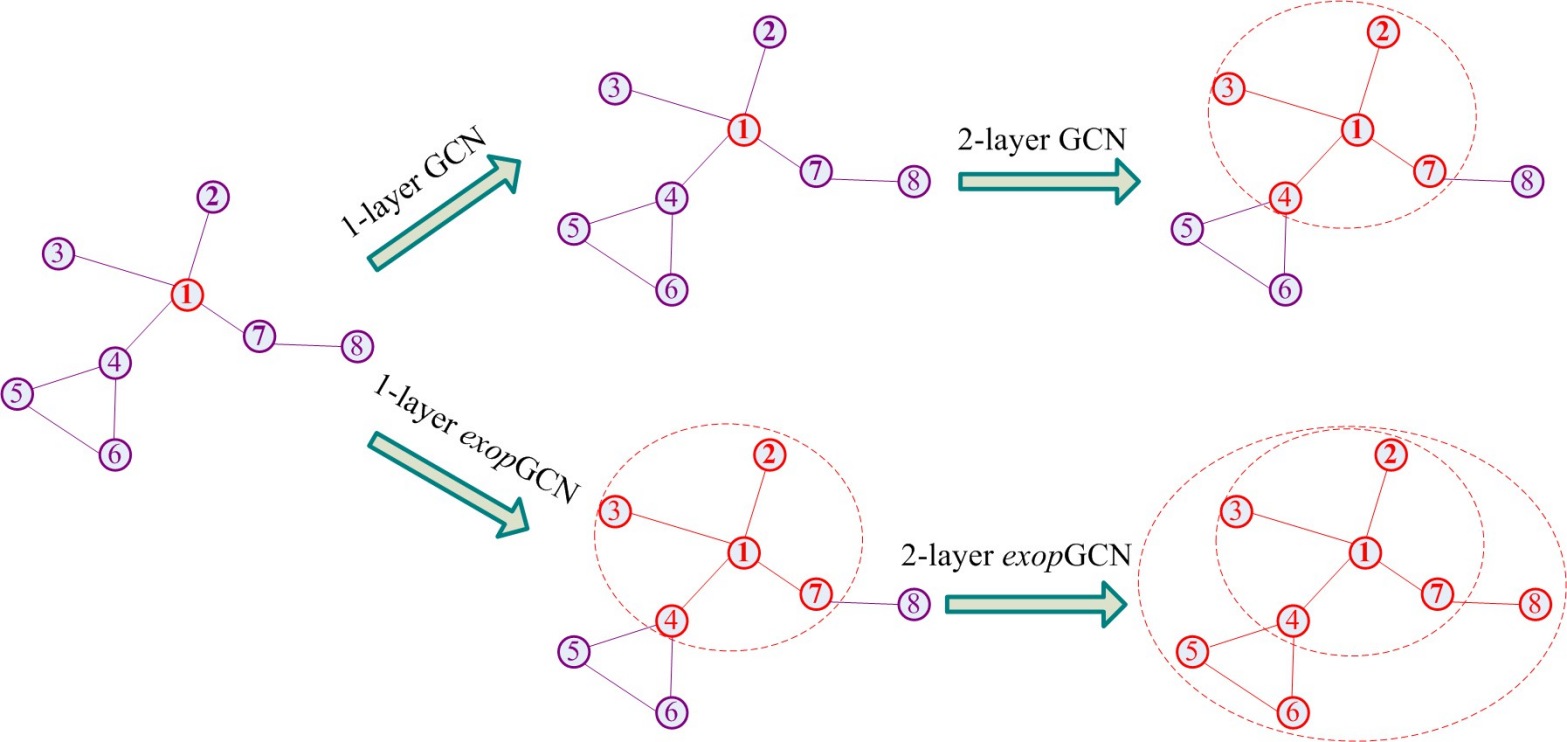
Comparison of GCN and *exop*GCN on acquiring information by neighboring nodes without features in this work, the feed forward propagation and softmax classifier of *exop*GCN are described as Eq (18) and Eq (19) respectively.


Using the extensional adjacency matrix **M**^*t*^, GCN can fuse the information of faster neighboring nodes in fewer layers. Take the node *v*_1_ as an example (see Fig 2), without node features, after 1-layer, GCN only contains its own information, and in 2-layer, GCN acquires the information from nodes *v*_2_,*v*_3_,*v*_4_ and *v*_7_ which are 1-path neighboring nodes. After 1-layer, *exop*GCN obtains the information from the nodes *v*_2_,*v*_3_,*v*_4_ and *v*_7_. After 2-layer, *exop*GCN acquires the information from all the nodes. Therefore, under the condition of the same number of layers, *exop*GCN can obtain more information from further nodes than GCN. As *t* increases, *exop*GCN quickly acquires information from more distant nodes.

$$H^{\left(l+1\right)}=\sigma ({\tilde{D^{t}}}^{-1/2}\tilde{M^{t}}{{\tilde{D^{t}}}^{-1/2}H}^{\left(l\right)}W^{\left(l\right)})$$ (18)

$$Z=softmax(\hat{M^{t}}ReLU(\hat{M^{t}}IW^{\left(0\right)})W^{\left(1\right)})$$ (19)

where $\tilde{M^{t}}=M^{t}+I,\tilde{D^{t}}$ is a degree matrix of $\tilde{M^{t}},\hat{M^{t}}={\tilde{D^{t}}}^{-1/2}\tilde{M^{t}}{\tilde{D^{t}}}^{-1/2}$.

## 3. Results

To evaluate the effectiveness of the proposed method *exop*GCN, we conducted empirical experiments on six publicly available datasets (see S1 Datasets), comparing its performance against 13 state-of-the-art GNN methods. These six datasets are Cora, Citeseer, Pubmed [28], Karate [24], Dolphins [29] and Polbook (http://www-personal.umich.edu/~mejn/netdata/, Books about US politics). Cora, Citeseer and Pubmed have 2708, 3312 and 19717 nodes, and 5429, 4732 and 44338 edges respectively. The nodes in the three graphs are divided into 7, 6 and 3 classes respectively. Note that, we only select the nodes with labels and features in Citeseer.

Karate, Dolphins and Polbook are small graphs with community structure, and the nodes in the three graphs do not have labels and features. They (Karate, Dolphins and Polbook) have 34, 62 and 105 nodes, and 78, 159 and 441 edges respectively. In order to evaluate the performance of different GNN methods, the nodes in the three graphs are divided by community labels. That is, we treat the nodes in the same community with the same class. As a result, Karate, Dolphins and Polbook are divided into 2, 2 and 3 classes respectively.

The performance of *exop*GCN is compared with other 13 GNN methods (The hyper-parameters setting for *exop*GCN and other GNNs is shown in S1 File). These 13 methods are GCN [6], FastGCN [11], GAT [30], SGC [31], ClusterGCN [12], DAGNN [32], APPNP [33], SSGC [34], GraphMLP [35], RobustGCN [36], LATGCN [37], MedianGCN [38] and ONF (ONFdw and ONFde) [18]. Some previous methods that deal with attribute-incomplete graphs have been proposed. But these methods, including SAT [19], GCNMF [20], D^2^PT [21], require some node features as input, and this is different from *exop*GCN method which does not require any node feature. Similar to *exop*GCN, the method (we abbreviate this method as ONF) proposed in the literature [18] does not need any node feature and classifies nodes by SGC [31]. The node features of ONF are generated by learning-based approaches and centrality-based approaches. Here, we first select two algorithms of node features generation with better performance [18, 23], that is deepwalk [39] (ONFdw) from learning-based approaches and degree (ONFde) from centrality-based approaches. The dimension of output node features for deepwalk is set to 64, and the node features generated by degree are represented by an one-hot vector [23]. Then, the performance on node classification of ONFdw and ONFde is compared with *exop*GCN.

In *exop*GCN, the convolutional operator **M**^*t*^ is generated by our work (see S2 Datasets), and the convolutional operator of other 13 GNNs are adjacency matrices **A**. Then, for Cora, Citeseer and Pubmed graphs (The indices of training and testing nodes for these three graphs are recorded in S1 File), we evaluate *exop*GCN and 13 GNN methods with 5% of the training size and 10% of the testing size, respectively. For other three small graphs, in order to improve performance, the training nodes are evenly selected from different communities (or labels) (The indices of training and testing nodes for three small graphs are recorded in S1 File). For example, in Karate with two communities (or labels), half of training nodes are from the first community, and remaining training nodes are from the other community. Since the sizes of the three graphs are small, we evaluate *exop*GCN and 13 GNN methods with 20% of the training size and 20% of the testing size, respectively. The accuracy of *exop*GCN and 13 GNN methods are shown in Table 1.

**Table 1:** The accuracy of different GNNs.

Methods	Cora	Citeseer	Pubmed	Karate	Dolphins	Polbook
*exop*GCN	**0.3741**	**0.4592**	**0.7855**	0.8333	**0.8462**	**0.9545**
GCN	0.1593	0.1722	0.7601	0.8333	0.3846	0.5455
FastGCN	0.2333	0.1390	0.7383	0.6667	0.6154	0.4545
GAT	0.3370	0.1208	0.7262	0.8333	0.3846	0.5000
SGC	0.3000	0.1118	0.6744	0.5000	0.7692	0.5909
ClusterGCN	0.3037	0.1752	0.7338	0.8333	0.5385	0.5000
DAGNN	0.2444	0.1601	0.7825	0.5000	0.3846	0.5000
APPNP	0.3148	0.1329	0.7368	0.6667	0.7692	0.5455
SSGC	0.2926	0.1329	0.7084	**1.0000**	0.3846	0.4545
GraphMLP	0.1630	0.2356	0.6410	0.6667	0.6154	0.4545
RobustGCN	0.2815	0.1118	0.6912	0.5000	0.3077	0.5000
LATGCN	0.1370	0.1420	0.7343	0.8333	0.3846	0.2727
MedianGCN	0.1556	0.1269	0.4777	0.5000	0.7962	0.4545
ONFdw	0.3148	0.1964	0.6699	**1.0000**	0.3846	0.5000
ONFde	0.3333	0.1390	0.4554	0.5000	0.4615	0.5000

The performance of *exop*GCN is improved significantly compared with other 13 GNNs on Cora, Citeseer, Pubmed, Dolphins and Polbook. The best performance is appeared on Polbook, the performance of *exop*GCN is better than that of worst-performing method (LATGCN) by 68.18% and is better than that of best-performing method (SGC) by 36.36%. Comparing with the worst-performing methods on Cora, Citeseer, Pubmed and Dolphins, the values of relative improvement of *exop*GCN are 23.71%, 34.74%, 33.01% and 53.85%, respectively. The values of relative improvement of *exop*GCN are 3.71%, 22.36%, 0.3% and 5% more than best-performing methods. *exop*GCN shows poor performance on Karate.

## 4. The performance of graph neural networks with the proposed convolutional operator

**Fig 3:**
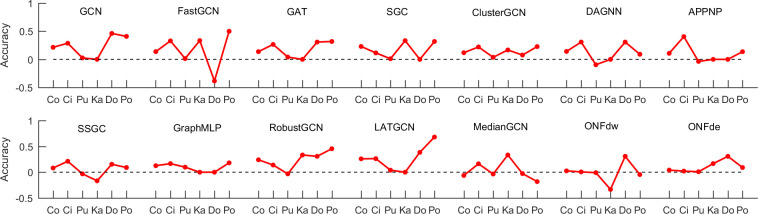
The improvement or decrease of accuracy for 13 GNNs using the proposed convolutional operator. Co, Ci, Pu, Ka, Do and Po represent Cora, Citeseer, Pubmed, Karate, Dolphins and Polbook.

In this section, we analyze the performance of current GNNs with the simple convolutional operator proposed in this work. First, 13 GNNs mentioned above are employed to test, and their convolutional operators (adjacency matrices) are replaced by our proposed convolutional operator (**M**^*t*^) (see S2 Datasets). Second, the 13 modified GNNs are used to classify nodes on the six graphs (Cora, Citeseer, Pubmed, Karate, Dolphins and Polbook). At last, the accuracy of 13 modified GNNs and original GNNs are computed. Note that, the parameter settings in this section are the same as those in the section of Results. Fig 3 shows the improvement or decrease of accuracy between the modified and original GNNs. From Fig 3, it can be observed that the accuracy of most GNNs are improved by using the proposed convolutional operator (**M**^*t*^) as a whole. Next, we investigate the results in detail. The accuracy of eight GNNs (GCN, FastGCN, GAT, SGC, ClusterGCN, GraphMLP, LATGCN and ONFde) are improved significantly on Cora, Citeseer, Pubmed and Polbook. One method (MedianGCN) obtains worse performance on Cora by adding the proposed convolutional operator. The relative digits are 0, 6, 2, 2 and 2 on Citeseet, Pubmed, Karate, Dolphins and Polbook, respectively. The best improvement of accuracy is LATGCN on the Poolbook by 68.18%. The worst performance is FastGCN with a decrement of 38.46% on Dolphins by adding the proposed convolutional operator. Generally speaking, these GNNs show poor performance on Pubmed probably because long-range propagation may bring redundant information for node classification. However, these results can provide one with a general skill to improve the accuracy of node classification without features.

## 5. Selection of the parameter *t*

**Fig 4:**
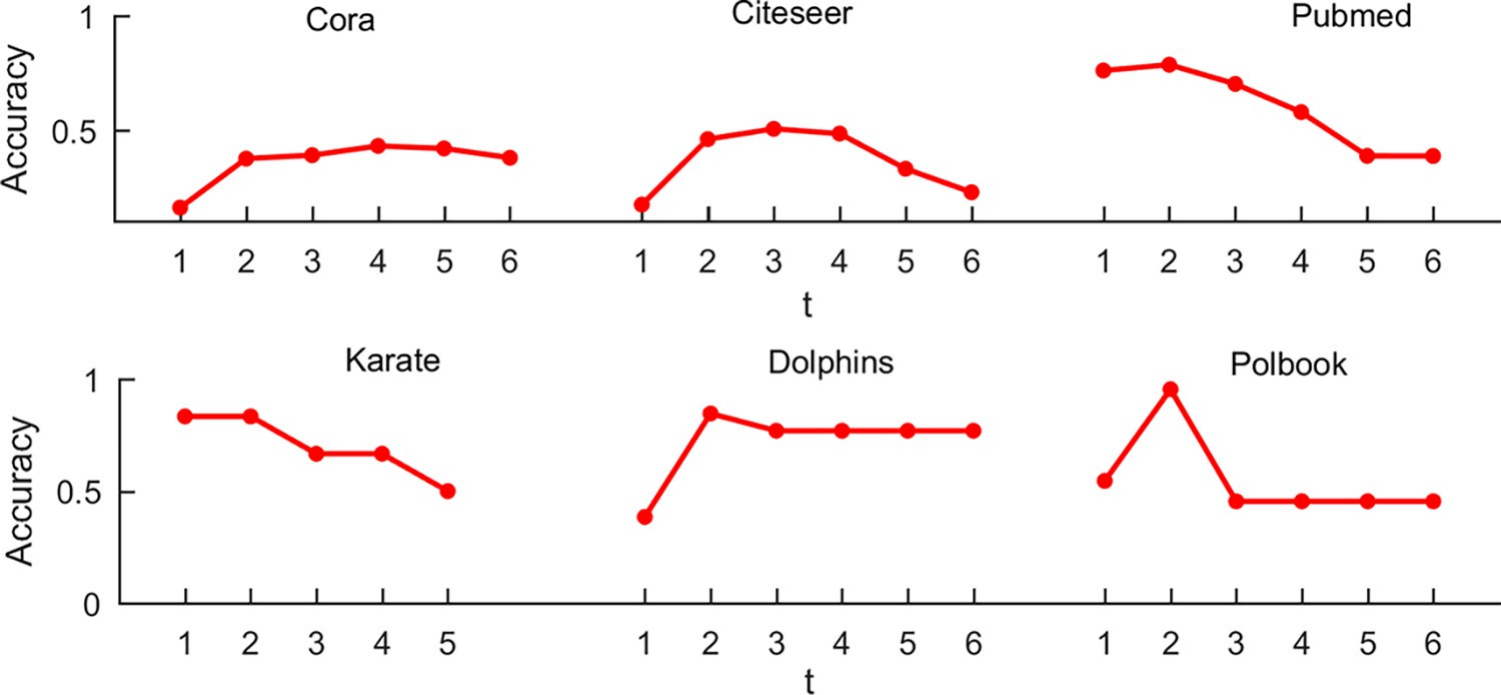
The relationship between the parameter *t* and the accuracy on the six graphs.

Selection of the parameter *t* plays a crucial role in improving the performance and preventing over fitting of *exop*GCN. Here, we discuss the relationship between the parameter *t* and the accuracy on the six graphs including Cora, Citeseer, Pubmed, Karate, Dolphins and Polbook. The parameter *t* is set from 1 to 6. Note that, if the graph diameter is less than 6, the upper limit of *t* is set to graph diameter. The results are show in Fig 4. From Fig 4, it can be observed that as the parameter *t* increase, the accuracy is reduced in general. On Pubmed, Karate, Dolphins and Polbook, the best accuracies appear when *t* is set to 2. For Citeseer, although the best accuracy with 50.45% is obtained when *t* is set to 3, *exop*GCN with *t* = 2 achieves a close accuracy of 45.92%. For Cora, the best accuracy with 42.96% is obtained when *t* is set to 4, and the accuracy is equal to 37.41% when *t* is set to 2. The phenomenon on Cora may be caused because it has diverse properties from other five graphs. In general, it is reasonable to set the parameter *t* to 2 for *exop*GCN, and the parameter with *t* = 2 can also prevent over fitting problem for diverse graphs.

## 6. The relationship between node classification and community detection

Furthermore, we discuss the relationship between node classification using GCN and community detection. From the Eq (13), it can be observed that GCN aggregates the information by the convolutional matrix and obtains similar information which has the same neighboring nodes when nodes do not have features. For example, the nodes 5 and 6 in Fig 1 have similar features. Therefore, GCN clusters the nodes with similar neighboring nodes into a class. The concept is close to the community structure in which the connections between nodes are tight, while the connections with other nodes in the network are loose [40].

In order to analyze the relationship between node classification using GCN and community detection, we first introduce edge (*e*_*ij*_) clustering coefficient [41] defined by Eq (20).


$$C_{ij}=\frac{z_{i,j}^{\left(3\right)}}{min[(k_{i}-1),(k_{j}-1)]}$$ (20)

In Eq (20), $z_{i,j}^{\left(3\right)}$ is the number of triangles of the edge *e*_*ij*_,*k*_*i*_ and *k*_*j*_ are the degree of nodes *v*_*i*_ and *v*_*j*_ respectively. Edge clustering coefficient also can be represented by neighboring nodes (see Eq (21)).


$$C_{ij}=\frac{\left|N_{ij}\right|}{min[(k_{i}-1),(k_{j}-1)]}$$ (21)

In Eq (21), *N*_*ij*_ is the common neighboring nodes set of the nodes *v*_*i*_ and *v*_*j*_.

From the definition of edge clustering coefficient in Eq (21), it can be observed that if nodes *v*_*i*_ and *v*_*j*_ have more common neighboring nodes, the edge clustering coefficient of the edge connecting node *v*_*i*_ and node *v*_*j*_ is greater. Likewise, we also find that GCN tends to cluster the two nodes with common neighboring nodes into a class because the two nodes have similar features. Therefore, if the edge clustering coefficient of the edge that connects node *v*_*i*_ and node *v*_*j*_ has large values, node *v*_*i*_ and node *v*_*j*_ are clustered into the same class by GCN. From the literature [41], we know that the edge (*e*_*ij*_) connecting node *v*_*i*_ and node *v*_*j*_ in the same community tends to have a large value of edge clustering coefficient. Therefore, if two nodes are grouped into a class by GCN, the two nodes are likely to be in the same community.

Modularity (see Eq (22)) [42] is widely used to measure community structure of a graph.


$$Q=\frac{1}{2m}\sum_{ij}\left(A_{ij}-P_{ij})\delta (C_{i},C_{j}\right)$$ (22)

In Eq (22), *m* and **A** represent the number of edges and the adjacency matrix respectively. **P**_*ij*_ is the expected number of edges between nodes *v*_*i*_ and *v*_*j*_ in the null model. δ = 1 if nodes *v*_*i*_ and *v*_*j*_ are in the same community (*C*_*i*_ = *C*_*j*_), zero otherwise.

In order to research the relationship between node classification using GCN and community detection, the accuracy of node classification using GCN and community detection are studied on both six real-world graphs (Cora, Citeseer, Pubmed, Karate, Dolphins and Polbook) and nine synthetic graphs with different values of modularity. The nine synthetic graphs called LFR benchmark are proposed by Lancichinetti et al [43]. In order to generate these synthetic graphs, some parameters should be set. (1) The number of nodes *n*, the average degree ⟨k⟩ and maximum degree ⟨max k⟩. (2) Minimum for the community sizes ⟨min c⟩ and maximum for the community sizes ⟨max c⟩. (3) Minus exponent for the degree sequence ⟨t1⟩ and minus exponent for the community size distribution ⟨t2⟩. (4) Mixing parameter ⟨μ⟩. The parameter μ is an index to represent community structure. Low μ indicates that the generated graphs have strong community structure. In this work, we set these parameters as follows: *n* = 1000, k = 8, max k = 40, t1 = 2, t2 = 1, min c = 5 and max c = 35. By turning the mixing parameter μ ∈ [0.1, 0.9] with a step 0.1, we will obtain 9 synthetic graphs (see S1 Datasets) with community labels.

**Fig 5:**
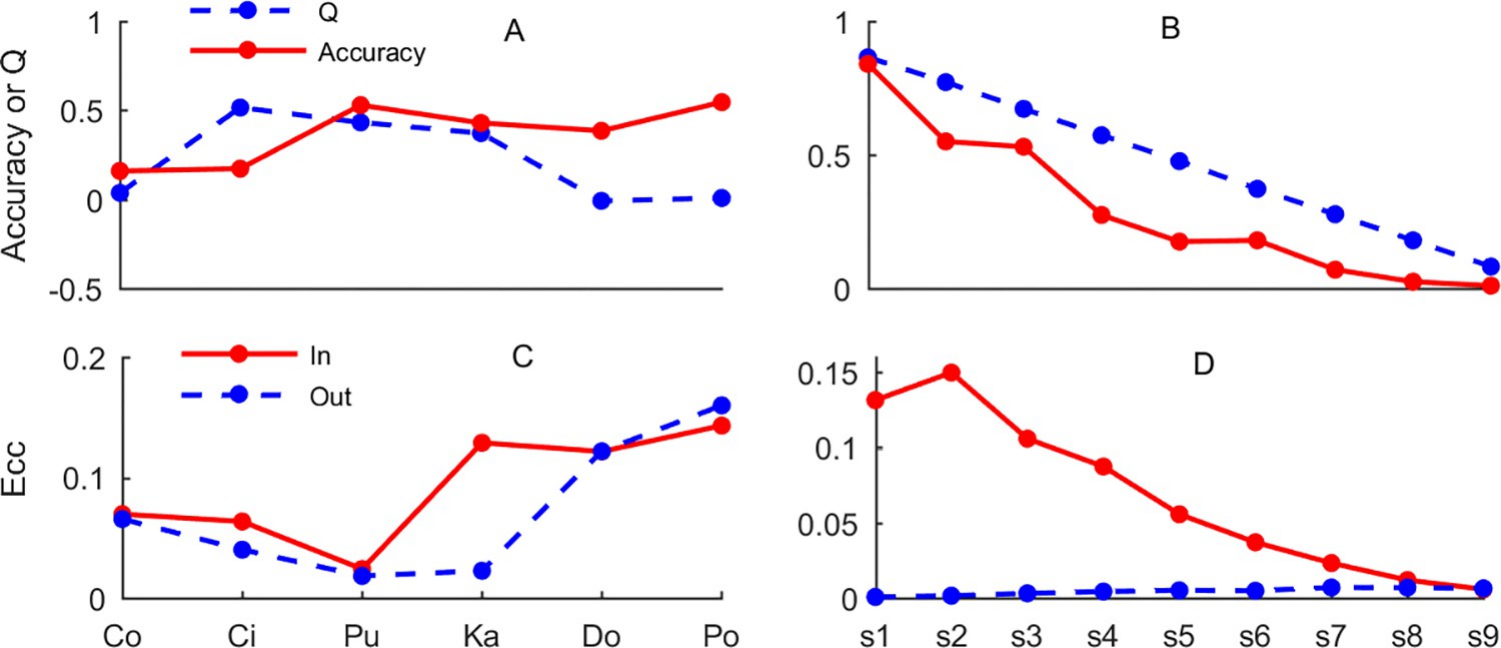
The relationship between accuracy of node classification using GCN and modularity Q. Ecc, In and Out represent edge clustering coefficient, $C_{in}^{m}$ and $C_{out}^{m}$, and s1, s2, s3, s4, s5, s6, s7, s8 and s9 represent nine synthetic graphs.

First, the nodes in the six real-world graphs (see S3 Datasets) and nine synthetic graphs (see S3 Datasets) are classed by GCN (The indices of training and testing nodes for these 15 graphs are recorded in S1 File). Note that, the training size and the testing size of six real-world graphs are set the same as in the section of Results. The training size and the testing size of nine synthetic graphs are set 20%. Second, the values of modularity Q are calculated by real labels (or community labels). At last, the accuracy of node classification using GCN and the values of Q are compared, and the results are shown in Fig 5. From Fig 5A, it can be observed that there is no strong regularity between the accuracy and the modularity Q overall. In detail, the high values of modularity Q means high values of accuracy on Pubmed and Karate. On the contrary, the relationship between accuracy and modularity Q has strong regularity (see Fig 5B) on nine synthetic graphs. It can be observed that the accuracy of GCN decreases with the decrease of Q value, this means that the principle of GCN and modularity Q may be similar, that is they tend to cluster the nodes with similar neighboring nodes into a class or a community.

We study the weak regularity in six real-world graphs and strong regularity in nine synthetic graphs in detail, and use a modified edge clustering coefficient (*C*^*m*^, see Eq (23)) to explain the phenomenon in Fig 5A and 5B. Here, we calculate two types of *C*^*m*^ of an edge *e*_*ij*_, that is $C_{in}^{m}$ of the edge in which the two nodes (*v*_*i*_ and *v*_*j*_) have the same label (or in the same community) and $C_{out}^{m}$ of the edge in which the two nodes (*v*_*i*_ and *v*_*j*_) have different labels (or in different communities). For an edge *e*_*ij*_, a high value of $C_{in}^{m}$ means that there are more common neighboring nodes between the two nodes (*v*_*i*_ and *v*_*j*_) connected by *e*_*ij*_, and two nodes can be classified by GCN with a high accuracy. While, a low value of $C_{out}^{m}$ means that there are fewer common neighboring nodes between the two nodes (*v*_*i*_ and *v*_*j*_) connected by *e*_*ij*_, and the edge *e*_*ij*_ can be easily broken by community detection methods with a high modularity Q. On the contrary, a high value of $C_{out}^{m}$ corresponds to a low value of modularity Q.

Therefore, we calculate the average values of $C_{in}^{m}$ and $C_{out}^{m}$ for each graphs, the results are shown in Fig 5C and 5D respectively. From the Fig 5, we observe that higher values of $C_{in}^{m}$ corresponds to higher accuracy on Cora, Citeseer, Karate, Dolphins and Polbook (see Fig 5A and 5C, solid red line) except for Pubmed. As previously analyzed, lower values of $C_{out}^{m}$ corresponds to higher modularity Q on citeseer, Pubmed and Karate (see Fig 5A and 5C, dotted blue line), and higher values of $C_{out}^{m}$ corresponds to lower modularity Q on Cora, Dolphins and Polbook (see Fig 5A and 5C, dotted blue line). Likewise, the phenomenon is appeared on eight synthetic graphs except for the second synthetic graphs. From the results mentioned above, the regularity between node classification using GCN and community detection (modularity Q) can be revealed by the modified edge clustering coefficient including $C_{in}^{m}$ and $C_{out}^{m}$.


$$C_{ij}^{m}=\frac{\left|N_{ij}\right|}{k_{i}+k_{j}}$$ (23)

## 7. Conclusion and discussion

Graph convolutional network (GCN), which represents node features by a convolutional matrix and propagation mechanisms, has become a power tool to deal with graph-structure data. But, the performance of GCN deteriorates when it encounters the graphs with missing node features. In order to resolve the problem, we first introduce a simple and effective convolutional operator by path-driven neighboring nodes, and then a modified GCN named *exop*GCN is proposed for node classification. Experimental results demonstrate that *exop*GCN show better performance for node classification on the graphs without node features comparing with other GNNs. Furthermore, the performance of 13 GNNs are improved significantly by adding the proposed convolutional operator, which means that our research can provide one with a general skill to improve the performance of GNNs for node classification on graphs without features. More important, using the edge clustering coefficient as a gap, the relationship between node classification using GCN without features and traditional community detection are researched. As a result, the positive relationship can reveal the mysterious theory of GCN from view of traditional and unsupervised methods.

Here, we discuss two issues of *exop*GCN and a direction of further research. The first issue is the application of *exop*GCN on node classification with features. To resolve the problem, *exop*GCN is employed to classify the nodes with features on Cora, Citeseer and Pubmed, and the results obtained by other 13 GNNs are provided for comparison (see S1 Table). As shown in S1 Table, compared to other 13 GNNs, *exop*GCN does not obtain the best performance on three graphs. This demonstrates that aggregation of features from long-range neighboring nodes does not improve the accuracy of *exop*GCN, but leads to redundancy of features, and thus cannot classify nodes effectively. The second issue is the complexity of *exop*GCN. Compared with GCN, the additional overhead is the computational cost of the convolutional operator **M**^2^. In fact, the computer of convolutional operator can be converted into K-hop reachability queries [44] with K = 2 for each nodes in the graphs, and many fast algorithms [45, 46] are proposed to resolve the problem and have been apply to large real-world graphs. Finally, although *exop*GCN achieves good performance on node classification without node feature, the performance of *exop*GCN and some GNNs deteriorates after adding the proposed convolutional operator (see Fig 3). The main reason for this phenomenon may be that the proposed convolutional operator carries redundant information. As with references [13, 14], we can use attention mechanism to select vital 2-path neighboring nodes and improve the performance of GNNs.

## Supporting information

S1 Datasets Six original graph data and nine synthetic graph data. (ZIP)

S2 Datasets Input data for *exop*GCN and different GNNs. (ZIP)

S3 Datasets Input data for GCN. (ZIP)

S1 File Parameters setting. (PDF)

S1 Table The accuracies of *exop*GCN and other GNNs on node classification with features. (PDF)
