# Supplementary material for: A simple and effective convolutional operator for node classification without features by graph convolutional networks
Source: PLoS One. 2024 Apr 30;19(4):e0301476. doi: 10.1371/journal.pone.0301476 (PMC11060547; doi:10.1371/journal.pone.0301476)
Supplement: S1 File — (PDF) [file pone.0301476.s004.pdf]

Parameters setting

1. The primary hyper-parameters in *exop*GCN and other GNNs. More detailed hyper-parameters can be obtained in GraphGallery. Note that these hyper-parameters are set by the GraphGallery which is a gallery for benchmarking graph neural networks. If you use these hyper-parameters, please cite: Li J, Xu K, Chen L, Zheng Z, Liu X. GraphGallery: A Platform for Fast Benchmarking and Easy Development of Graph Neural Networks Based Intelligent Software. 2021 IEEE/ACM 43rd International Conference on Software Engineering: Companion Proceedings.

***exop*GCN**

Learning rate: 0.01

Maximum epochs: 100

Dropout rate: 0.5

L2 regularization weight: 5e-4

Hidden units: 16

GCN

Learning rate: 0.01

Maximum epochs: 100

Dropout rate: 0.5

L2 regularization weight: 5e-4

Hidden units: 16

FastGCN

Learning rate: 0.01

Maximum epochs: 100

Dropout rate: 0.5

L2 regularization weight: 5e-4

Hidden units: 32

GAT

Learning rate: 0.005

Maximum epochs: 100

Dropout rate: 0.6

L2 regularization weight: 5e-4

Hidden units: 8

SGC

Learning rate: 0.2

Maximum epochs: 100

Dropout rate: 0.5

L2 regularization weight: 5e-6

Hidden units: 32

ClusterGCN

Learning rate: 0.01

Maximum epochs: 100

Dropout rate: 0.5

Hidden units: 32

L2 regularization weight: 0

DAGNN

Learning rate: 0.01

Maximum epochs: 100

Dropout rate: 0.5

L2 regularization weight: 5e-3

Hidden units: 64

APPNP

Learning rate: 0.01

Maximum epochs: 100

Dropout rate: 0.5

L2 regularization weight: 5e-4

Hidden units: 64

SSGC

Learning rate: 0.2

Maximum epochs: 100

Dropout rate: 0.5

L2 regularization weight: 5e-5

Hidden units: 32

GraphMLP

Learning rate: 0.001

Maximum epochs: 100

Dropout rate: 0.6

L2 regularization weight: 5e-3

Hidden units: 256

RobustGCN

Learning rate: 0.01

Maximum epochs: 100

Dropout rate: 0.5

L2 regularization weight: 5e-4

Hidden units: 64

LATGCN

Learning rate: 0.01

Maximum epochs: 100

Dropout rate: 0.2

L2 regularization weight: 5e-4

Hidden units: 16

MedianGCN

Learning rate: 0.01

Maximum epochs: 100

Dropout rate: 0.5

L2 regularization weight: 1e-4

Hidden units: 16

ONF (This method classifies nodes by SGC)

Learning rate: 0.2

Maximum epochs: 100

Dropout rate: 0.5

L2 regularization weight: 5e-6

Hidden units: 32

2. The node indices for training, validation and testing. Note that the node indices for training [0:134] means that the nodes at positions 0 to 134 in the 'node_order' file serve as the training set. The 'node_order' file for each graph is obtained in S3 Datasets.

For Cora

Node indices for training: [0:134]

Node indices for validation: [500:600]

Node indices for testing: [1000:1270]

For Citeseer

Node indices for training: [0:165]

Node indices for validation: [500:600]

Node indices for testing: [1000:1331]

For Pubmed

Node indices for training: [0:986]

Node indices for validation: [5000:5600]

Node indices for testing: [10000:11972]

For karate

Node indices for training: [0:6]

Node indices for validation: [7:9]

Node indices for testing: [25:31]

For Dolphins

Node indices for training: [0:12]

Node indices for validation: [50:56]

Node indices for testing: [25:38]

For Polbook

Node indices for training: [0:22]

Node indices for validation: [50:61]

Node indices for testing: [70:92]

For nine synthetic graphs

Node indices for training: [0:199]

Node indices for validation: [500:600]

Node indices for testing: [700:900]
