# Supplementary material for: A simple and effective convolutional operator for node classification without features by graph convolutional networks
Source: PLoS One. 2024 Apr 30;19(4):e0301476. doi: 10.1371/journal.pone.0301476 (PMC11060547; doi:10.1371/journal.pone.0301476)
Supplement: S1 Table — (PDF) [file pone.0301476.s005.pdf]

S1 Table The accuracies of *exop*GCN and other GNNs on node classification with features

Methods	Cora	Citeseer	Pubmed
*exop*GCN	0.3630	0.3051	0.8078
GCN	0.3481	0.3323	0.8367
FastGCN	0.3444	0.2991	0.8540
GAT	0.3444	0.3505	0.8367
SGC	0.3407	0.2387	0.6714
ClusterGCN	0.3111	0.4713	0.8367
DAGNN	0.3556	0.5498	**0.8545**
APPNP	0.3741	0.4834	0.8433
SSGC	0.3370	0.2598	0.8068
GraphMLP	**0.5519**	**0.6073**	0.8200
RobustGCN	0.3333	0.1511	0.3854
LATGCN	0.3259	0.2719	0.7885
MedianGCN	0.3000	0.2961	0.8088
ONFdw	0.3148	0.1964	0.6699
ONFde	0.3333	0.1390	0.4554
